# 2264. Shorter Duration of Visit Associated with Inappropriate Antibiotic Prescribing for Respiratory Tract Infections in a National Sample of Ambulatory Visits, 2009 - 2016

**DOI:** 10.1093/ofid/ofad500.1886

**Published:** 2023-11-27

**Authors:** Vidal M Mendoza, Kelsey Strey, Kelly Reveles

**Affiliations:** UT College of Pharmacy, San Antonio, Texas; The University of Texas at Austin College of Pharmacy, San Antonio, Texas; The University of Texas at Austin, San Antonio, Texas

## Abstract

**Background:**

Due to the reported decrease in the duration of primary care visits, it is important to assess provider adherence to guidelines when treating respiratory infections that are unlikely to necessitate antimicrobial treatment. This study aimed to investigate the relationship between the duration of visit and unnecessary antibiotic prescribing while considering patient and provider factors, including age, sex, race, and diagnosis.

**Methods:**

This retrospective, cross-sectional study used outpatient, office-based data from the National Ambulatory Medical Care Survey from 2009 to 2016. All patients with a primary diagnosis of bronchitis, viral respiratory tract infection, influenza, and viral pneumonia were included for analysis. Patients with diagnoses that almost always require an antibiotic were excluded. Diagnoses were identified using ICD-9 or ICD-10 codes. Inappropriate antibiotic use was identified using multum codes and defined as at least one prescribed oral antibiotic during the visit. Provider status was defined as physician only or other provider. Multivariate logistic regression was used to determine predictors of inappropriate antibiotic prescribing. Data weights were applied to all analyses to generate national estimates.

**Results:**

A total of 226,250,589 patient visits were included in the analysis, of which 83,404,484 (36.9%) resulted in an inappropriately prescribed antibiotic. Patients prescribed antibiotics were older (median age 42 vs. 13 years) and more often White (82.4% vs. 79.6%). After adjustment for covariates, time spent with provider less than 15 minutes (aOR 1.866, 95% CI 1.862-1.869), patient age 18-64 years (aOR 2.408, 95% CI 2.406-2.410), other provider types only (aOR 1.790, 95% CI 1.785-1.794), and viral pneumonia diagnosis (aOR 68.432, 95% CI 66.691-69.936) were most strongly associated with inappropriate antibiotic prescribing.

**Conclusion:**

This study found that patients who spent less time with providers, had viral pneumonia, and were aged 18-64 years old were more likely to receive inappropriate treatment. Further investigation is necessary to determine reasons for inappropriate antimicrobial prescribing among outpatients.

**Disclosures:**

**Kelly Reveles, PharmD, PhD**, Ferring Pharmaceuticals: Advisor/Consultant|Ferring Pharmaceuticals: Honoraria

